# Beneficial Effect of Antioxidants in Retinopathies: A New Hypothesis

**Published:** 2012

**Authors:** Isabella Panfoli

**Affiliations:** Department of Pharmacy-DIFAR, Biochemistry Lab. University of Genoa, Genova, Italy

**Keywords:** Antioxidants, Retinopathy, Oxidative stress

## Abstract

The retina is the most oxygen consuming tissue of the body. Rod and cone photoreceptors efficiently carry out visual cascades, which are energetically costly processes. Data has recently been published that suggests that the metabolic support to phototransduction in the rod outer segment (OS) may originate directly in the OS, which is able to conduct aerobic metabolism. This oxygen-handling activity of the rod OS, which was never suspected before, appears to be a primary cause of the generation of reactive oxygen species directly inside the OS. Oxidative stress has been hypothesised to contribute to most of the neurodegenerative retinal pathologies, such as diabetic retinopathy, age-related macular degeneration, retinitis pigmentosa and photoreceptor cell death after retinal detachment. Many natural antioxidant compounds are routinely used in experimental or human therapies for preventing or delaying photoreceptor degeneration in those pathologies. Here it is proposed that the ultimate reason for the beneficial actions of antioxidants in preventing or retarding the effect on the retinal degenerative pathologies can be found in their action on reactive oxygen species generated by the ectopic mitochondrial electron transport chain (ETC) coupled to FoF1-ATP synthase in rod OS disks. In fact, if not adequately coupled, the ETC generates reactive oxygen species that, in turn, can act on the polyunsaturated fatty acids which the rod OS is rich in. If correct, the mechanism put forward here would provide a potential for the molecular basis of therapies with antioxidants for retinal degenerative diseases.

## INTRODUCTION

The vertebrate retina contains two types of photoreceptors, rods and cones, which carry out the first step of vision. Both possess a specialised compartment: the outer segment (OS) that is dedicated to phototransduction, and the inner segment (IS), which contains the subcellular organelles and nucleus. The retina is particularly susceptible to oxidative stress because of its high O2 consumption [1], its high proportion of polyunsaturated fatty acids [2, 3], and its exposure to visible light. Considering the limited understanding of the origin of the ATP supply in OS [4-6], where continual ATP consumption is observed particularly during light adaptation, a high cGMP flux rate is observed [7] (i.e. a higher steady PDE activity). In addition, there is a correspondingly higher activity of guanylate cyclase, the enzyme that synthesises cyclic GMP from GTP. Moreover, it was shown that visual transduction is supported by oxidative metabolism [8], and that anaerobic glycolysis is not sufficient to provide enough ATP in the light. This group has conducted proteomic analysis of purified OS disks and reported [9] that these express most of the subunits of the mitochondrial machinery for oxidative phosphorylation, such as Kreb’s cycle enzymes and electron transport chain (ETC) proteins, as well as FoF1-ATP synthase. The ETC complexes are composed of NADH dehydrogenase (ETC I), succinate dehydrogenase (ETC II), ubiquinol-cytochrome c oxidoreductase (ETC III), cytochrome c, cytochrome c oxidase (ETC IV) and F1Fo-ATP synthase (V), performing the oxidative phosphorylation that is currently believed to be exclusive to the mitochondrial membrane in eukaryotes. Rod disks possess active respiratory complexes that may build up a proton gradient also in vivo, which the ectopic ATP synthase can utilise to aerobically synthesise ATP outside the mitochondrion. The OS would supply chemical energy for phototransduction through extra-mitochondrial aerobic ATP synthesis [4, 9-11]. Similar results were obtained on intact myelin vesicles [12-14] and C6 glioma cell plasma membranes [15]. The activity of the Tricarboxylic Acid (TCA) cycle enzymes was also reported to be consistent in the rod OS [11] , which is in keeping with the knowledge that many mitochondrial proteins possess dual or multiple localisations, [16] and that mitochondria are dynamic organelles [17]. While the mitochondrial proteome consists of more than 1,000 different proteins, many proteomic analyses of cellular membranes have found the exclusive expression of proteins from the five respiratory complexes [18]. Therefore, a large proportion of the high retinal rate of O2 consumption would depend on the rod OS, justifying the phenomenon of rod-induced hypoxia on a quantitative basis [6]. Taking into consideration the existence of O2 consumption in the rod OS, a hypothesis for the involvement of oxidative stress in the pathogenic mechanism for many acquired and inherited retinal degenerations has been recently proposed [6]. Moreover, a decline in the rod OS ETC functioning, along with an increase in reactive oxygen species production and the consequent chronic oxidative stress, would generate hypometabolism, in turn causing an imbalance in the clearance of proteins. This may cause the aggregation of peptides and the generation of drusen [6]. The ETC embedded in the disk membranes would be a primary source of superoxides [19]. Reactive oxygen species are in fact by-products of ETC functioning, and their overproduction is foreseen in any pathological condition that uncouples ATP synthase from the ETC. 

Oxidative stress has been implicated in the development of diabetic retinopathy, and has also been shown to be a risk factor for age-related macular degeneration (AMD) [20- and retinitis pigmentosa (RP) [24-26], which are the most common degenerative diseases of the retina. It was also shown to play a fundamental role in photoreceptor cell death after retinal detachment (RD) [27]. Moreover, extensive research suggests that, in those retinal pathologies, naturally occurring compounds with antioxidant actions, that are components of a normal diet, are greatly beneficial. Several studies showed that antioxidants appeared to retard or inhibit the degenerative pathology [28-30]. Interestingly, curcumin, anthocianins and cathechins are inhibitors of ATP synthase [31].

## HYPOTHESES

Considering the aforementioned facts, the present paper hypothesises that the reason for the efficacy of antioxidants in preventing or retarding the onset of many retinal degenerative pathologies in both experimental and clinical studies is a result of their scavenging action on reactive oxygen species produced inside the OS. These species can be generated by the ectopically-expressed mitochondrial ETC that is coupled to ATP synthase in rod OS disks. In fact, it is quite doubtful that antioxidants can scavenge reactive oxygen species inside the mitochondrion, as they have been found to be unable to penetrate mitochondria [31].

## DISCUSSION

Oxidative stress is also implicated in the development of diabetic complications, in particular diabetic retinopathy [32]. Moreover, the antioxidant mechanisms are impaired in diabetic conditions [33-35]. The administration of antioxidants to diabetic rats prevents oxidative damage in the retina and the development of retinopathy [36]. Lutein prevented the impairment of the electroretinogram observed in controls [37]. The antioxidant lutein, a dietary carotenoid, was also shown to be useful in AMD [28]. The Eye Disease Case-Control Study found that high plasma levels of lutein and zeaxanthin are associated with a reduced risk of neovascular AMD [38, 39].

 Oxidative stress plays an important role in photoreceptor cell death after RD. Treatment with a reactive oxygen species scavenger (e.g. Edaravone) was shown to prevent photoreceptor cell death after RD [27]. The model of oxidative stress in photoreceptor cell death after RD is interesting as it rules out a role for retinal pigmented epithelium. Oxidative stress may also act as a mediator of retinal degeneration in RP, and is, in fact, believed to play an important role in photoreceptor cell death [40, 41]. Increased lutein dietary intake increases macular pigment optical density, improving visual function [42]. Anthocyanins are known to be effective ingredients for maintaining eye health, as they are potent antioxidants that have been shown to exert a role against lipid peroxidation [43] and to protect against retinal damage [44, 45]. Extensive research suggests that pro-anthocyanins are beneficial to health in general, especially in improving vision, because of their antioxidant effects [46]. Anthocyanin-rich bilberry extract prevented the impairment of photoreceptor cell function, as measured by electroretinogram, in a mouse model of endotoxin-induced uveitis [47]. Catechins, and EGCG in particular, act as biological antioxidants [48], which are able to scavenge superoxide, hydroxyl radicals, singlet oxygen and peroxynitrite [46, 48]. Curcumin (1,7-bis(4-hydroxy-3-methoxyphenyl)1,6-heptadiene-3,5-dione), which is a principal component of turmeric, is a promising candidate for the rescue of retinal ischemic diseases [31, 49]. Curcumin is well known for its anti-tumour, antioxidant, and anti-inflammatory properties. Its anti-protein aggregating activity was examined in particular studies on rats expressing the P23H rhodopsin (Rh) mutation, and it rescued photoreceptors from degeneration due to the toxicity of Rh aggregates [49]. 

Oxidative stress, i.e. the cellular damage caused by reactive oxygen species (free radicals, superoxide, hydrogen peroxide, and singlet oxygen), has been hypothesised to contribute to the development of AMD [23, 39], and diabetic retinopathy [28, 50], which are among the most common cause of blindness in the United States. The retina is considered to be particularly susceptible to oxidative stress, and the contribution of the rod OS should be considered [6] because of its unexpected O2 consumption ability [1], and the high proportion of polyunsaturated fatty acids in the disk membranes [2], which are highly enriched in docosahexaenoic acid (DHA). DHA is prone to peroxidation, one of the major events induced by oxidative stress, particularly in polyunsaturated fatty acid-rich biomembranes. The photoreceptor OS contain the highest concentration of polyunsaturated fatty acids of any vertebrate tissue, and, in fact, is considered a model for the study of lipid peroxidation [3]. Furthermore, there is strong evidence suggesting that lipofuscin, which is produced during oxidative damage and is a photo-reactive substance, is found in the photoreceptor OS as a consequence of oxidative stress [51]. It was also shown that complex I in particular is the major complex responsible for the generation of reactive oxygen species [19]. In turn, disk membrane lipid peroxidation would be associated with impairment of the protein functions therein located, in particular the ETC, which are extremely sensitive to the state of the membrane environment. Moreover, the disruption of cytochromes might free iron, thereby generating dangerous redox reactions. 

## CONCLUSION

The extensive literature data on the protective role of antioxidants utilised in many pharmaceutical preparations for humans, on photoreceptor degeneration, as well as several reports of a pivotal role of oxidative stress in the pathogenesis of degenerating retinal diseases, is suggestive of a role of oxygen radicals on the OS itself. This appears easier to understand when considering the expression of a functional ETC in OS disks, which is an efficient but dangerous location for O2 utilisation, especially considering the presence of polyunsaturated fatty acids. 

## DISCLOSURE

The authors report no conflicts of interest in this work.
